# Do Experiences With Nature Promote Learning? Converging Evidence of a Cause-and-Effect Relationship

**DOI:** 10.3389/fpsyg.2019.00305

**Published:** 2019-02-19

**Authors:** Ming Kuo, Michael Barnes, Catherine Jordan

**Affiliations:** ^1^Landscape and Human Health Laboratory, Department of Natural Resources and Environmental Sciences, University of Illinois at Urbana-Champaign, Urbana, IL, United States; ^2^Department of Forest Resources, University of Minnesota, Saint Paul, MN, United States; ^3^Department of Pediatrics, University of Minnesota, Minneapolis, MN, United States; ^4^Children & Nature Network, Minneapolis, MN, United States

**Keywords:** literature review, green space, instruction, teaching, environmental education, nature-based learning, green schoolyard

## Abstract

Do experiences with nature – from wilderness backpacking to plants in a preschool, to a wetland lesson on frogs—promote learning? Until recently, claims outstripped evidence on this question. But the field has matured, not only substantiating previously unwarranted claims but deepening our understanding of the cause-and-effect relationship between nature and learning. Hundreds of studies now bear on this question, and converging evidence strongly suggests that experiences of nature boost academic learning, personal development, and environmental stewardship. This brief integrative review summarizes recent advances and the current state of our understanding. The research on personal development and environmental stewardship is compelling although not quantitative. Report after report – from independent observers as well as participants themselves – indicate shifts in perseverance, problem solving, critical thinking, leadership, teamwork, and resilience. Similarly, over fifty studies point to nature playing a key role in the development of pro-environmental behavior, particularly by fostering an emotional connection to nature. In academic contexts, nature-based instruction outperforms traditional instruction. The evidence here is particularly strong, including experimental evidence; evidence across a wide range of samples and instructional approaches; outcomes such as standardized test scores and graduation rates; and evidence for specific explanatory mechanisms and active ingredients. Nature may promote learning by improving learners’ attention, levels of stress, self-discipline, interest and enjoyment in learning, and physical activity and fitness. Nature also appears to provide a calmer, quieter, safer context for learning; a warmer, more cooperative context for learning; and a combination of “loose parts” and autonomy that fosters developmentally beneficial forms of play. It is time to take nature seriously as a resource for learning – particularly for students not effectively reached by traditional instruction.

## Introduction

The intuition that “nature is good for children” is widely held, and yet, historically, the evidence for this intuition has been uncompelling, with a distressing number of weak studies and inflated claims. Now, however, an impressive body of work has accrued and converging lines of evidence paint a convincing picture.

This integrative mini-review (see Supplementary Material for methods) summarizes what we know about the role of nature experiences in learning and development. It draws on a wide array of peer-reviewed scientific evidence, ranging from research in the inner city, to the study of Attention Deficit/Hyperactivity Disorder, to neurocognitive and physiological explorations. Our overarching question was, “do nature experiences promote learning and child development?”

Throughout our review, we took care to distinguish between evidence for cause-and-effect relationships and evidence for associations; causal language (e.g., “affects,” “boosts,” “is reduced by”) is used only where justified by experimental evidence. Where converging, but not experimental, evidence points to a likely cause-and-effect relationship, our language is qualified accordingly (e.g., “seems to increase”). Table 1 summarizes recent advances in this area and explains how those advances contribute to our confidence in a cause-and-effect relationship between nature and learning and development.

**Table 1:** Do nature experiences promote learning? Advances in methodology and evidence.

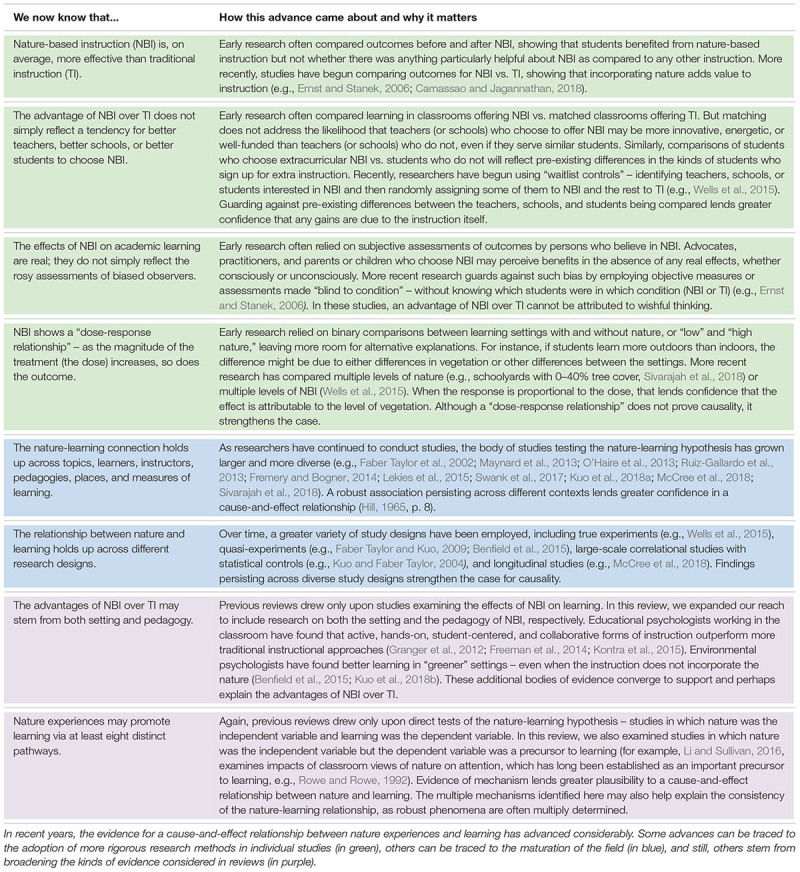

**FIGURE 1:**
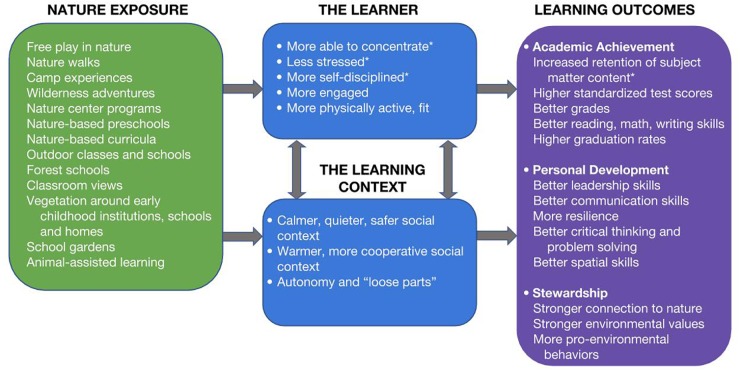
Nature-based learning: exposures, probable mechanisms, and outcomes. This Figure summarizes the state of the scientific literature on nature and learning. The items and pathways here emerged from our review as opposed to guiding our review; thus each item listed has been empirically associated with one or more other items in the figure. Relationships for which there is cause-and-effect evidence are indicated with an asterisk; for example, “more able to concentrate” is asterisked because experimental research has demonstrated that exposure to nature boosts concentration. Similarly, “increased retention of subject matter” is asterisked because experimental research has demonstrated that exposure to nature in the course of learning boosts retention of that material. Here and throughout this review, causal language (e.g., “affects,” “increases,” “boosts,” “is reduced by”) is used only where experimental evidence (the gold standard for assessing cause-and-effect) warrants. Where converging evidence suggests a causal relationship but no experimental evidence is available, we use qualified causal language (e.g., “seems to increase”). The green box lists forms of nature exposure that have been tied with learning, whether directly (nature -> learning) or indirectly, via one or more of the mechanisms listed (nature -> mechanism -> learning). In this review, “nature” includes experiences of nature not only in wilderness but also within largely human-made contexts. Thus a classroom with a view of trees offers an experience of nature not offered by its counterpart facing the school parking lot. This review encompassed experiences of nature regardless of context – whether through play, relaxation, or educational activities, and in informal, non-formal and formal settings. The blue boxes show probable mechanisms – intermediary variables which have been empirically tied to both nature and learning. For example, the ability to concentrate is rejuvenated by exposure to nature and plays an important role in learning. Natural settings may affect learning both by directly fostering a learner’s capacity to learn and by providing a more supportive context for learning. The purple box lists learning outcomes that have been tied to contact with nature. In this review, “learning” encompasses changes in knowledge, skills, behaviors, attitudes, and values. A database of articles found in the three phases of the review process (ending in 2018) is available at: https://goo.gl/FZ1CA9.

What emerged from this critical review was a coherent narrative (Figure 1): experiences with nature do promote children’s academic learning and seem to promote children’s development as persons and as environmental stewards – and at least eight distinct pathways plausibly contribute to these outcomes. Below, we discuss the evidence for each of the eight pathways and then the evidence tying nature to learning, personal development, and the development of stewardship.

## Nature May Boost Learning Via Direct Effects on Learners

Five of the eight plausible pathways between nature and learning we identified are centered in the learner. Learning is likely to improve when a learner is more attentive (Rowe and Rowe, 1992; Mantzicopoulos and Morrison, 1994); less stressed (Grannis, 1992; Leppink et al., 2016); more self-disciplined (Mischel et al., 1988; Duckworth and Seligman, 2005); more engaged and interested (Taylor et al., 2014 for review); and more physically active and fit (for reviews, see Álvarez-Bueno et al., 2017; Santana et al., 2017). Evidence suggests that contact with nature contributes to each of these states or conditions in learners.

### Nature Has Rejuvenating Effects on Attention

The rejuvenating effect of nature on mentally fatigued adults (e.g., Hartig et al., 1991; Kuo, 2001) and children has been demonstrated in a large body of studies, including field experiments (Faber Taylor and Kuo, 2009) and large-scale longitudinal studies (Dadvand et al., 2015). Students randomly assigned to classrooms with views of greenery perform better on concentration tests than those assigned to purely “built” views or windowless classrooms (Li and Sullivan, 2016). Nature’s rejuvenating effects on attention have been found in students going on field trips (van den Berg and van den Berg, 2011), Swedish preschoolers (Mårtensson et al., 2009), children in Chicago public housing (Faber Taylor et al., 2002), and 5 to 18-year-olds with ADHD (e.g., Kuo and Faber Taylor, 2004), using measures of attention ranging from parent and teacher ratings (O’Haire et al., 2013) to neurocognitive tests (Schutte et al., 2015).

### Nature Relieves Stress

The stress-reducing effects of nature have been documented in adults in a large body of controlled experiments (see Kuo, 2015; Supplementary Material for review) and the available evidence points to a similar effect in children. Nature has been related to lower levels of both self-reported and physiological measures of stress in children (Bell and Dyment, 2008; Chawla, 2015; Wiens et al., 2016). Recently, an experimental study showed that a window view of vegetation from a high school classroom yields systematic decreases in heart rate and self-reported stress, whereas built views do not (Li and Sullivan, 2016). Further, students learning in a forest setting one day a week showed healthier diurnal rhythms in cortisol in that setting than a comparison group that learned indoors – cortisol dropped over the course of the school day when lessons were held in the forest but not in the classroom – and these effects could not be attributed to the physical activity associated with learning outdoors (Dettweiler et al., 2017).

### Contact With Nature Boosts Self-Discipline

In adults, the effects of viewing scenes of nature on self-discipline have been demonstrated experimentally using tests of impulse control (Berry et al., 2014; Chow and Lau, 2015). In children, nature contact has been tied to greater self-discipline in children from inner city Chicago (Faber Taylor et al., 2002) to residential Barcelona (Amoly et al., 2014) and in experimental (Sahoo and Senapati, 2014), longitudinal (Ulset et al., 2017), and large-scale cross-sectional studies (Amoly et al., 2014). These benefits have been shown for neurotypical children as well as for children with ADHD (Sahoo and Senapati, 2014) and learning difficulties (Ho et al., 2017). The types of self-discipline assessed include delay of gratification (Faber Taylor et al., 2002) and parent ratings of hyperactivity (Flouri et al., 2014), and the types of “nature” include not just “greenness” but contact with horses in animal-assisted learning (Ho et al., 2017). Note that impulse control effects are not always statistically significant (e.g., Amoly et al., 2014; Schutte et al., 2015). Nonetheless, in general, impulse control is better during or after children’s contact with nature.

### Student Motivation, Enjoyment, and Engagement Are Better in Natural Settings

Student motivation, enjoyment, and engagement are better in natural settings, perhaps because of nature’s reliably positive effects on mood (e.g., Takayama et al., 2014). In previous reviews (Blair, 2009; Becker et al., 2017) and recent studies (e.g., Skinner and Chi, 2014; Alon and Tal, 2015; Lekies et al., 2015), students and teachers report strikingly high levels of student engagement and motivation, during both student-elected and school-mandated nature activities. Importantly, learning in and around nature is associated with intrinsic motivation (Fägerstam and Blom, 2012; Hobbs, 2015), which, unlike extrinsic motivation, is crucial for student engagement and longevity of interest in learning. The positivity of learning in nature seem to ripple outward, as seen in learners’ engagement in subsequent, indoor lessons (Kuo et al., 2018a), ratings of course curriculum, materials, and resources (Benfield et al., 2015) and interest in school in general (Blair, 2009; Becker et al., 2017), as well as lower levels of chronic absenteeism (MacNaughton et al., 2017). Encouragingly, learning in nature may improve motivation most in those students who are least motivated in traditional classrooms (Dettweiler et al., 2015).

### Time Outdoors Is Tied to Higher Levels of Physical Activity and Fitness

While the evidence tying green space to physical activity is extremely mixed (see Lachowitz and Jones, 2011 for review), children’s time outdoors is consistently tied to both higher levels of physical activity and physical fitness: the more time children spend outdoors, the greater their physical activity, the lesser their sedentary behavior, and the better their cardiorespiratory fitness (Gray et al., 2015). Importantly, cardiorespiratory fitness is the component of physical fitness most clearly tied to academic performance (Santana et al., 2017). Further, there is some indication greener school grounds can counter children’s trend toward decreasing physical activity as they approach adolescence: in one study, girls with access to more green space and woodlands, and boys with access to ball fields, were more likely to remain physically active as they got older (Pagels et al., 2014). This pattern is echoed in later life: in older adults, physical activity declines with age – but among those living in greener neighborhoods the decline is smaller (Dalton et al., 2016).

## Nature May Boost Learning by Providing a More Supportive Context for Learning

In addition to its effects on learners, natural settings and features may provide a more supportive context for learning in at least three ways. Greener environments may foster learning because they are calmer and quieter, because they foster warmer relationships, and because the combination of “loose parts” and relative autonomy elicits particularly beneficial forms of play.

### Vegetated Settings Tend to Provide Calmer, Quieter, Safer Contexts for Learning

Both formal and informal learning are associated with a greater sense of calmness or peace when conducted in greener settings (Maynard et al., 2013; Nedovic and Morrissey, 2013; Chawla et al., 2014). Problematic and disruptive behaviors such as talking out of turn or pushing among children are less frequent in natural settings than in the classroom (Bassette and Taber-Doughty, 2013; Nedovic and Morrissey, 2013; O’Haire et al., 2013; Chawla et al., 2014). Further, in greener learning environments, students who previously experienced difficulties in traditional classrooms are better able to remove themselves from conflicts and demonstrate better self-control (Maynard et al., 2013; Ruiz-Gallardo et al., 2013; Swank et al., 2017). The social environment of the classroom has long been recognized as important for learning (Rutter, 2000). Calmer environments have been tied to greater student engagement and academic success (Wessler, 2003; McCormick et al., 2015).

### Natural Settings Seem to Foster Warmer, More Cooperative Relations

Images of nature have prosocial effects in adults (e.g., Weinstein et al., 2009) and greener settings are tied to the development of meaningful and trusting friendships between peers (White, 2012; Chawla et al., 2014; Warber et al., 2015). Maynard et al. (2013) theorize that natural settings provide a less restrictive context for learning than the traditional classroom, giving children more freedom to engage with one another and form ties. Indeed, learning in greener settings has been consistently tied to the bridging of both socio-cultural differences and interpersonal barriers (e.g., personality conflicts) that can interfere with group functioning in the classroom (White, 2012; Cooley et al., 2014; Warber et al., 2015). Finally, learning in nature facilitates cooperation and comfort between students and teachers, perhaps by providing a more level playing-field wherein the teacher is seen as a partner in learning (Scott and Colquhoun, 2013). More cooperative learning environments promote student engagement and academic performance (Patrick et al., 2007; McCormick et al., 2015).

### Natural Settings May Afford “Loose Parts,” Autonomy, and Distinctly Beneficial Forms of Play

In his “theory of loose parts,” Nicholson (1972) posited that the “stuff” of nature – sticks, stones, bugs, dirt, water – could promote child development by encouraging creative, self-directed play. Indeed, teachers’ and principals’ observations suggest children’s play becomes strikingly more creative, physically active, and more social in the presence of loose parts (e.g., Bundy et al., 2008, 2009). Interestingly, it appears that nature, loose parts, and autonomy can each independently contribute to outcomes (see Bundy et al., 2009; Niemiec and Ryan, 2009; Studente et al., 2016, respectively), raising the possibility of synergy among these factors. Although the effects of loose parts play on child development have yet to be quantitatively demonstrated (Gibson et al., 2017), the potential contributions of more creative, more social, more physically active play to cognitive, social and physical development seem clear.

## Outcomes for Learning and Development

In school settings, incorporating nature in instruction improves academic achievement over traditional instruction. In a randomized controlled trial of school garden-based instruction involving over 3,000 students, students gained more knowledge than waitlist control peers taking traditional classes; moreover, the more garden-based instruction, the larger the gains (Wells et al., 2015). Further, among the over 200 other tests of nature-based instruction’s academic outcomes, the vast majority of findings are positive (for reviews, see Williams and Dixon, 2013; Becker et al., 2017) – and here, too, the most impressive findings come from studies employing the largest doses of nature-based instruction (e.g., Ernst and Stanek, 2006). Findings have been consistently positive across diverse student populations, academic subjects, instructors and instructional approaches, educational settings, and research designs.

Interestingly, both the pedagogy and setting of nature-based instruction may contribute to its effects. Hands-on, student-centered, activity-based and discussion-based instruction each outperform traditional instruction—even when conducted indoors (Granger et al., 2012; Freeman et al., 2014; Kontra et al., 2015). And simply conducting traditional instruction in a more natural setting may boost outcomes. In multiple studies, the greener a school’s surroundings, the better its standardized test performance – even after accounting for poverty and other factors (e.g., Sivarajah et al., 2018)—and classrooms with green views yield similar findings (Benfield et al., 2015; although c.f. Doxey et al., 2009). The frequency of positive findings on nature-based instruction likely reflects the combination of a better pedagogy and a better educational setting.

In and outside the context of formal instruction, experiences of nature seem to contribute to additional outcomes. First, not only do experiences of nature enhance academic learning, but they seem to foster personal development – the acquisition of intrapersonal and interpersonal assets such as perseverance, critical thinking, leadership, and communication skills. While quantitative research on these outcomes is rare, the qualitative work is voluminous, striking, and near-unanimous (for reviews, see Cason and Gillis, 1994; Williams and Dixon, 2013; Becker et al., 2017). Teachers, parents, and students report that wilderness and other nature experiences boost self-confidence, critical thinking, and problem-solving (e.g., Kochanowski and Carr, 2014; Truong et al., 2016) as well as leadership and communication skills such as making important decisions, listening to others, and voicing opinions in a group (e.g., Jostad et al., 2012; Cooley et al., 2014). Students emerge more resilient, with a greater capacity to meet challenges and thrive in adverse situations (Beightol et al., 2012; Cooley et al., 2014; Harun and Salamuddin, 2014; Warber et al., 2015; Richmond et al., 2017). Interestingly, greener everyday settings may also boost positive coping (Kuo, 2001) and buffer children from the impacts of stressful life events (Wells and Evans, 2003).

And second, spending time in nature appears to grow environmental stewards. Adults who care strongly for nature commonly attribute their caring to time, and particularly play, in nature as children – and a diverse body of studies backs them up (for review, see Chawla and Derr, 2012). Interestingly, the key ingredient in childhood nature experiences that leads to adult stewardship behavior does not seem to be conservation knowledge (knowledge of how and why to conserve). Although knowledge of how and why to conserve, which could presumably be taught in a classroom setting, has typically been assumed to drive stewardship behavior, it is relatively unimportant in predicting conservation behavior (Otto and Pensini, 2017). By contrast, an emotional connection to nature, which may be more difficult to acquire in a classroom, is a powerful predictor of children’s conservation behavior, explaining 69% of the variance (Otto and Pensini, 2017). Indeed, environmental attitudes may foster the acquisition of environmental knowledge (Fremery and Bogner, 2014) rather than vice versa. As spending time in nature fosters an emotional connection to nature and, in turn, conservation attitudes and behavior, direct contact with nature may be the most effective way to grow environmental stewards (Lekies et al., 2015).

## Conclusion and Implications

Do experiences with nature really promote learning? A scientist sampling some of the studies in this area might well be dismayed initially – as we were – at the frequency of weak research designs and overly optimistic claims. But a thorough review reveals an evidence base stronger, deeper, and broader than this first impression might suggest: weak research designs are supplemented with strong ones; striking findings are replicated in multiple contexts; the research on nature and learning now includes evidence of mechanisms; and findings from entirely outside the study of nature and learning point to the same conclusions.

Robust phenomena are often robust because they are multiply determined. The eight likely pathways between exposure to nature and learning identified here may account for the consistency of the nature-learning connection. Certainly it seems likely that increasing a student’s ability to concentrate, interest in the material, and self-discipline simultaneously would enhance their learning more than any of these effects alone. Moreover, in a group setting, effects on individual learners improve the learning context; when Danika fidgets less, her seatmates Jamal and JiaYing experience fewer disruptions and concentrate better; when Danika, Jamal, and JiaYing are less disruptive, the whole class learns better. These synergies – within and between students – may help explain how relatively small differences in schoolyard green cover predict significant differences in end-of-year academic achievement performance (e.g., Matsuoka, 2010; Kuo et al., 2018b).

An important question arose in the course of our review: is nature-based instruction effective for students for whom traditional instruction is ineffective? Although this review was not structured to systematically assess this question, the benefits of nature-based learning for disadvantaged students were a striking leitmotif in our reading. Not only can nature-based learning work better for disadvantaged students (McCree et al., 2018; Sivarajah et al., 2018), but it appears to boost interest in uninterested students (Dettweiler et al., 2015; Truong et al., 2016), improve some grades (Camassao and Jagannathan, 2018), and reduce disruptive episodes and dropouts among “at risk” students (Ruiz-Gallardo et al., 2013). Nature-based learning may sometimes even erase race- and income-related gaps (e.g., Taylor et al., 1998). Further, anecdotes abound in which students who ordinarily struggle in the classroom emerge as leaders in natural settings. If nature is equigenic, giving low-performing students a chance to succeed and even shine, the need to document this capacity is pressing. In the United States, where sixth graders in the richest school districts are four grade levels ahead of children in the poorest districts (Reardon et al., 2017), this need is urgent.

Fully assessing and making use of the benefits of nature-based instruction can serve all children. The available evidence suggests that experiences of nature help children acquire some of the skills, attitudes, and behaviors most needed in the 21st century. “Non-cognitive factors” such as perseverance, self-efficacy, resilience, social skills, leadership, and communication skills – so important in life beyond school (National Research Council, 2012) – are increasingly recognized by the business community and policy makers as essential in a rapidly changing world. And for generations growing up in the Anthropocene, environmental stewardship may be as important as any academic content knowledge.

We conclude it is time to take nature seriously as a resource for learning and development. It is time to bring nature and nature-based pedagogy into formal education – to expand existing, isolated efforts into increasingly mainstream practices. Action research should assess the benefits of school gardens, green schoolyards and green walls in classrooms. Principals and school boards should support, not discourage, teachers’ efforts to hold classes outdoors, take regular field trips, and partner with nearby nature centers, farms, and forest preserves. Teachers who have pioneered nature-based instruction should serve as models and coaches, helping others address its challenges and take full advantage of its benefits.

## Author Contributions

All authors co-wrote and edited the manuscript. MK provided leadership for decisions of content, framing, and style and led the creation of the Figure and Table. MB created the SoNBL literature database on which this review is based. CJ serves as the principal investigator of the Science of Nature-Based Learning Collaborative Research Network project; in addition to initiating this project and substantially shaping the Figure and Table, she solicited feedback from Network members.

## Conflict of Interest Statement

The authors declare that the research was conducted in the absence of any commercial or financial relationships that could be construed as a potential conflict of interest.

## References

[B1] Alon N. L. Tal T. (2015). Student self-reported learning outcomes of field trips: the pedagogical impact. *Int. J. Sci. Educ.* 37 1279–1298. 10.1080/09500693.2015.1034797

[B2] Álvarez-Bueno C. Pesce C. Cavero-Redondo I. Sánchez-López M. Garrido-Miguel M. Martínez-Vizcaíno V. (2017). Academic achievement and physical activity: a meta-analysis. *Pediatrics* 140:e20171498. 10.1542/peds.2017-1498 29175972

[B3] Amoly E. Dadvand P. Forns J. López-Vicente M. Basagaña X. Julvez J. (2014). Green and blue spaces and behavioral development in Barcelona schoolchildren: the breathe project. *Environ. Health Perspect.* 122 1351–1358. 10.1289/ehp.1408215 25204008 PMC4256702

[B4] Bassette L. A. Taber-Doughty T. (2013). The effects of a dog reading visitation program on academic engagement behavior in three elementary students with emotional and behavioral difficulties: a single case design. *Child Youth Care Forum* 42 239–256. 10.1007/s10566-013-9197-y

[B5] Becker C. Lauterbach G. Spengler S. Dettweiler U. Mess F. (2017). Effects of regular classes in outdoor education settings: a systematic review on students’ learning, social and health dimensions. *Int. J. Environ. Res. Public Health* 14:E485. 10.3390/ijerph14050485 28475167 PMC5451936

[B6] Beightol J. Jevertson J. Gray S. Carter S. Gass M. A. (2012). Adventure education and resilience enhancement: a mixed methods study. *J. Exp. Educ.* 35 307–325.

[B7] Bell A. C. Dyment J. E. (2008). Grounds for health: the intersection of school grounds and health-promoting schools. *Environ. Educ. Res.* 14 77–90. 10.1080/13504620701843426

[B8] Benfield J. A. Rainbolt G. N. Bell P. A. Donovan G. H. (2015). Classrooms with nature views: evidence of different student perceptions and behaviors. *Environ. Behav.* 47 140–157. 10.1177/0013916513499583

[B9] Berry M. S. Sweeney M. M. Morath J. Odum A. L. Jordan K. E. (2014). The nature of impulsivity: visual exposure to natural environments decreases impulsive decision-making in a delay discounting task. *PLoS One* 9:e97915. 10.1371/journal.pone.0097915 24841421 PMC4026519

[B10] Blair D. (2009). The child in the garden: an evaluative review of the benefits of school gardening. *J. Environ. Educ.* 40 15–38. 10.3200/JOEE.40.2.15-38

[B11] Bundy A. C. Luckett T. Naughton G. A. Tranter P. J. Wyver S. R. Ragen J. (2008). Playful interaction: occupational therapy for all children on the school playground. *Am. J. Occup. Ther.* 62 522–527. 10.5014/ajot.62.5.522 18826012

[B12] Bundy A. C. Luckett T. Tranter P. J. Naughten G. A. Wyver S. R. Ragen J. (2009). The risk is that there is ”no risk”: a simple innovative intervention to increase children’s activity levels. *Int. J. Early Years Educ.* 17 33–45. 10.1080/09669760802699878

[B13] Camassao M. J. Jagannathan R. (2018). Nature thru nature: creating natural science identities in populations of disadvantaged children through community education partnership. *J. Environ. Educ.* 49 30–42. 10.1080/00958964.2017.1357524

[B14] Cason D. Gillis H. L. (1994). A meta-analysis of outdoor adventure programming with adolescents. *J. Exp. Educ.* 17 40–47. 10.1177/105382599401700109

[B15] Chawla L. (2015). Benefits of nature contact for children. *J. Plan. Lit.* 30 433–452. 10.1177/0885412215595441

[B16] Chawla L. Derr V. (2012). “The development of conservation behaviors in childhood and youth,” in *The Oxford Handbook of Environmental and Conservation Psychology*, ed. Clayton S. D. (Oxford: Oxford University Press), 527–555.

[B17] Chawla L. Keena K. Pevec I. Stanley E. (2014). Green schoolyards as havens from stress and resources for resilience in childhood and adolescence. *Health Place* 28 1–13. 10.1016/j.healthplace.2014.03.001 24691122

[B18] Chow J. T. Lau S. (2015). Nature gives us strength: exposure to nature counteracts ego-depletion. *J. Soc. Psychol.* 155 70–85. 10.1080/00224545.2014.972310 25310796

[B19] Cooley S. J. Holland M. J. G. Cumming J. Novakovic E. G. Burns V. E. (2014). Introducing the use of a semi-structured video diary room to investigate students’ learning experiences during an outdoor adventure education groupwork skills course. *High. Educ.* 67 105–121. 10.1007/s10734-013-9645-5

[B20] Dadvand P. Niewenhuijsen M. J. Esnaola M. Forns J. Basagaña X. Alvarez-Pedrero M. (2015). Green spaces and cognitive development in primary schoolchildren. *Proc. Natl. Acad. Sci.U.S.A.* 112 7937–7942. 10.1073/pnas.1503402112 26080420 PMC4491800

[B21] Dalton A. M. Wareham N. Griffin S. Jones A. P. (2016). Neighbourhood greenspace is associated with a slower decline in physical activity in older adults: a prospective cohort study. *SSM Popul. Health* 2 683–691. 10.1016/j.ssmph.2016.09.006 28018960 PMC5165047

[B22] Dettweiler U. Becker C. Auestad B. H. Simon P. Kirsch P. (2017). Stress in school. Some empirical hints on the circadian cortisol rhythm of children in outdoor and indoor classes. *Int. J. Environ. Res. Public Health* 14:475. 10.3390/ijerph14050475 28468292 PMC5451926

[B23] Dettweiler U. Ünlü A. Lauterbach G. Becker C. Gschrey B. (2015). Investigating the motivational behavior of pupils during outdoor science teaching within self-determination theory. *Front. Psychol.* 6:125. 10.3389/fpsyg.2015.00125 25741301 PMC4331641

[B24] Doxey J. Waliczek T. M. Zajicek J. M. (2009). The impact of interior plants in university classrooms on student course performance and on student perceptions of the course and instructor. *Hortic. Sci.* 44 384–391. 10.21273/HORTSCI.44.2.384

[B25] Duckworth A. L. Seligman M. E. P. (2005). Self-discipline outdoes IQ in predicting academic performance of adolescents. *Psychol. Sci.* 16 939–944. 10.1111/j.1467-9280.2005.01641.x 16313657

[B26] Ernst J. Stanek D. (2006). The prairie science class: a model for re-visioning environmental education within the national wildlife refuge system. *Hum. Dimens. Wildl.* 11 255–265. 10.1080/10871200600803010

[B27] Faber Taylor A. Kuo F. Sullivan W. (2002). Views of nature and self-discipline: evidence from inner city children. *J. Environ. Psychol.* 22 49–63. 10.1006/jevp.2001.0241

[B28] Faber Taylor A. Kuo F. E. (2009). Children with attention deficits concentrate better after walk in the park. *J. Atten. Disord.* 12 402–409. 10.1177/1087054708323000 18725656

[B29] Fägerstam E. Blom J. (2012). Learning biology and mathematics outdoors: effects and attitudes in a Swedish high school context. *J. Advent. Educ. Outdoor Learn.* 13 56–75. 10.1080/14729679.2011.647432

[B30] Flouri E. Midouhas E. Joshi H. (2014). The role of urban neighbourhood green space in children’s emotional and behavioural resilience. *J. Environ. Psychol.* 40 179–186. 10.1016/j.jenvp.2014.06.007 28495219

[B31] Freeman S. Eddy S. L. McDonough M. Smith M. K. Okoroafor N. Jordt H. (2014). Active learning increases student performance in science, engineering, and mathematics. *PNAS* 111 8410–8415. 10.1073/pnas.1319030111 24821756 PMC4060654

[B32] Fremery C. Bogner F. X. (2014). Cognitive learning in authentic environments in relation to green attitude preferences. *Stud. Educ. Eval.* 44 9–15. 10.1016/j.stueduc.2014.11.002

[B33] Gibson J. L. Cornell M. Gill T. (2017). A systematic review of research into the impact of loose parts play on children’s cognitive, social and emotional development. *School Ment. Health* 9 295–309. 10.1007/s12310-017-9220-9 29170683 PMC5680404

[B34] Granger E. M. Bevis T. H. Saka Y. Southerland S. A. Sampson V. Tate R. L. (2012). The efficacy of student-centered instruction in supporting science learning. *Science* 338 105–108. 10.1126/science.1223709 23042893

[B35] Grannis J. C. (1992). Students’ stress, distress, and achievement in an urban intermediate school. *J. Early Adolesc.* 12 4–27. 10.1177/0272431692012001001

[B36] Gray C. Gibbons R. Larouche R. Sandseter E. B. Bienenstock A. Brussoni M. (2015). What is the relationship between outdoor time and physical activity, sedentary behaviour, and physical fitness in children? A systematic review. *Int. J. Environ. Res. Public Health* 12 6455–6474. 10.3390/ijerph120606455 26062039 PMC4483711

[B37] Hartig T. Mang M. Evans G. W. (1991). Restorative effects of natural environmental experiences. *Environ. Behav.* 23 3–26. 10.1177/0013916591231001

[B38] Harun M. T. Salamuddin N. (2014). Promoting social skills through outdoor education and assessing its effects. *Asian Soc. Sci.* 10 71–78. 10.5539/ass.v10n5p71

[B39] Hill A. B. (1965). The Environment and Disease: Association or Causation? *Proc. R. Soc. Med.* 58 295–300. 14283879 10.1177/003591576505800503 PMC1898525

[B40] Ho N. F. Zhou J. Fung D. S. S. Kua P. H. J. Huang Y. X. (2017). Equine-assisted learning in youths at-risk for school or social failure. *Cogent Educ.* 4:1334430 10.1080/2331186X.2017.1334430

[B41] Hobbs L. K. (2015). Play-based science learning activities: engaging adults and children with informal science learning for preschoolers. *Sci. Commun.* 37 405–414. 10.1177/1075547015574017

[B42] Jostad J. Paisley K. Gookin J. (2012). Wilderness-based semester learning: understanding the NOLS experience. *J. Outdoor Recreat. Educ. Leadersh.* 4 16–26. 10.7768/1948-5123.1115

[B43] Kochanowski L. Carr V. (2014). Nature playscapes as contexts for fostering self-determination. *Chil. Youth Environ.* 24 146–167. 10.7721/chilyoutenvi.24.2.0146

[B44] Kontra C. Lyons D. J. Fischer S. M. Beilock S. L. (2015). Physical experience enhances learning. *Psychol. Sci.* 26 737–749. 10.1177/0956797615569355 25911125

[B45] Kuo F. E. (2001). Coping with poverty: impacts of environment and attention in the inner city. *Environ. Behav.* 33 5–34. 10.1177/00139160121972846

[B46] Kuo F. E. Faber Taylor A. (2004). A potential natural treatment for attention-deficit/hyperactivity disorder: evidence from a national study. *Am. J. Public Health* 94 1580–1586. 10.2105/AJPH.94.9.1580 15333318 PMC1448497

[B47] Kuo M. (2015). How might contact with nature promote human health? Promising mechanisms and a possible central pathway. Supplemental material. *Front. Psychol.* 6:1093. 10.3389/fpsyg.2015.01093 26379564 PMC4548093

[B48] Kuo M. Browning M. H. E. M. Penner M. L. (2018a). Do lessons in nature boost subsequent classroom engagement? Refueling students in flight. *Front. Psychol.* 8:2253. 10.3389/fpsyg.2017.02253 29354083 PMC5758746

[B49] Kuo M. Browning M. H. E. M. Sachdeva S. Westphal L. Lee K. (2018b). Might school performance grow on trees? Examining the link between “greenness” and academic achievement in urban, high-poverty schools. *Front. Psychol.* 9:1669. 10.3389/fpsyg.2018.01669 30319478 PMC6168033

[B50] Lachowitz K. Jones A. P. (2011). Greenspace and obesity: a systematic review of the evidence. *Obes. Rev.* 12 e183–e189. 10.1111/j.1467-789X.2010.00827.x 21348919

[B51] Lekies K. S. Lost G. Rode J. (2015). Urban youth’s experiences of nature: implications for outdoor adventure education. *J. Outdoor Recreat. Tour.* 9 1–10. 10.1016/j.jort.2015.03.002

[B52] Leppink E. W. Odlaug B. L. Lust K. Christenson G. Grant J. E. (2016). The young and the stressed: stress, impulse control, and health in college students. *J. Nervous Ment. Disord.* 204 931–938. 10.1097/NMD.0000000000000586 27575792

[B53] Li D. Sullivan W. C. (2016). Impact of views to school landscapes on recovery from stress and mental fatigue. *Landsc. Urban Plan.* 148 149–158. 10.1016/j.landurbplan.2015.12.015

[B54] MacNaughton P. Eitland E. Kloog I. Schwartz J. Allen J. (2017). Impact of particulate matter exposure and surrounding “greenness” on chronic absenteeism in Massachusetts public schools. *Int. J. Environ. Res. Public Health* 14:E207. 10.3390/ijerph14020207 28230752 PMC5334761

[B55] Mantzicopoulos P. Y. Morrison D. (1994). A comparison of boys and girls with attention problems: kindergarten through second grade. *Am. J. Orthopsychiatry* 64 522–533. 10.1037/h0079560 7847568

[B56] Mårtensson F. Boldemann C. Soderstrom M. Blennow M. Englund J.-E. Grahn P. (2009). Outdoor environmental assessment of attention promoting settings for preschool children. *Health Place* 15 1149–1157. 10.1016/j.healthplace.2009.07.002 19643655

[B57] Matsuoka R. H. (2010). Student performance and high school landscapes: examining the links. *Landsc. Urban Plan.* 97 273–282. 10.1016/j.landurbplan.2010.06.011

[B58] Maynard T. Waters J. Clement C. (2013). Child-initiated learning, the outdoor environment and the “underachieving” child. *Early Years* 33 212–225. 10.1080/09575146.2013.771152

[B59] McCormick M. P. Cappella E. O’Conner E. E. McClowry S. G. (2015). Social-emotional learning and academic achievement: using causal methods to explore classroom-level mechanisms. *AERA Open* 1 1–26. 10.1177/233285841560395928164148

[B60] McCree M. Cutting R. Sherwin D. (2018). The hare and the tortoise go to forest school: taking the scenic route to academic attainment via emotional wellbeing outdoors. *Early Child Dev. Care* 188 980–996. 10.1080/03004430.2018.1446430

[B61] Mischel W. Shoda Y. Peake P. (1988). The nature of adolescent competencies predicted by preschool delay of gratification. *J. Pers. Soc. Psychol.* 54 687–696. 10.1037/0022-3514.54.4.687 3367285

[B62] National Research Council. (2012). *Education for Life and Work: Developing Transferable Knowledge and Skills in the 21st Century*. Washington, DC: The National Academies Press.

[B63] Nedovic S. Morrissey A. (2013). Calm, active and focused: children’s responses to an organic outdoor learning environment. *Learn. Environ. Res.* 16 281–295. 10.1007/s10984-013-9127-9

[B64] Nicholson S. (1972). The theory of loose parts, an important principle for design methodology. *Stud. Des. Educ. Craft Technol.* 4 5–14.

[B65] Niemiec C. P. Ryan R. M. (2009). Autonomy, competence, and relatedness in the classroom: applying self-determination theory to educational practice. *Theory Res. Educ.* 7 133–144. 10.1177/1477878509104318

[B66] O’Haire M. E. Mckenzie S. J. McCune S. Slaughter V. (2013). Effects of animal-assisted activities with guinea pigs in the primary school classroom. *Anthrozoos* 26 455–458. 10.2752/175303713X13697429463835 24265514 PMC3832256

[B67] Otto S. Pensini P. (2017). Nature-based environmental education of children: environmental knowledge and connectedness to nature, together, are related to ecological behaviour. *Glob. Environ. Change* 47 88–94. 10.1016/j.gloenvcha.2017.09.009

[B68] Pagels P. Raustorp A. Ponce De Leon A. Mårtensson F. Kylin M. Boldemann C. (2014). A repeated measurement study investigating the impact of school outdoor environment upon physical activity across ages and seasons in Swedish second, fifth and eighth graders. *Biomed Central Public Health* 14:803. 10.1186/1471-2458-14-803 25099142 PMC4133613

[B69] Patrick H. Ryan A. M. Kaplan A. (2007). Early adolescents’ perceptions of the classroom social environment, motivational beliefs, and engagement. *J. Educ. Psychol.* 99 83–98. 10.1037/0022-0663.99.1.83

[B70] Reardon S. F. Kalogrides D. Shores K. (2017). *The Geography of Racial/Ethnic Test Score Gaps (CEPA Working Paper No.16-10)*. Available at: Stanford Center for Education Policy Analysis: http://cepa.stanford.edu/wp16-10

[B71] Richmond D. Sibthorp J. Gookin J. Annorella S. Ferri S. (2017). Complementing classroom learning through outdoor adventure education: out-of-school-time experiences that make a difference. *J. Advent. Educ. Outdoor Learn.* 18 1–17.

[B72] Rowe K. J. Rowe K. S. (1992). The relationship between inattentiveness in the classroom and reading achievement: part B: an explanatory study. *J. Am. Acad. Child Adolesc. Psychiatry* 31 357–368. 10.1097/00004583-199203000-00026 1564039

[B73] Ruiz-Gallardo J. Verde A. Valdes A. (2013). Garden-based learning: an experience with “at risk” secondary education students. *J. Environ. Educ.* 44 252–270. 10.1080/00958964.2013.786669 19559139

[B74] Rutter M. (2000). “School effects on pupil progress: research findings and policy implications,” in *Psychology of Education: Major Themes* Vol. 1 eds Smith P. K. Pellegrini A. D. (London: Falmer Press), 3–150.

[B75] Sahoo S. K. Senapati A. (2014). Effect of sensory diet through outdoor play on functional behaviour in children with ADHD. *Indian J. Occup. Ther.* 46 49–54.

[B76] Santana C. C. A. Azevedo L. B. Cattuzzo M. T. Hill J. O. Andrade L. P. Prado W. L. (2017). Physical fitness and academic performance in youth: a systematic review. *Scand. J. Med. Sci. Sports* 27 579–603. 10.1111/sms.12773 27714852

[B77] Schutte A. R. Torquati J. C. Beattie H. L. (2015). Impact of urban nature on executive functioning in early and middle childhood. *Environ. Behav.* 49 3–30. 10.1177/0013916515603095

[B78] Scott G. Colquhoun D. (2013). Changing spaces, changing relationships: the positive impact of learning out of doors. *Aust. J. Outdoor Educ.* 17 47–53. 10.1007/BF03400955

[B79] Sivarajah S. Smith S. M. Thomas S. C. (2018). Tree cover and species composition effects on academic performance of primary school students. *PLoS One* 13:e0193254. 10.1371/journal.pone.0193254 29474503 PMC5825089

[B80] Skinner E. A. Chi U. (2014). Intrinsic motivation and engagement as “active ingredients” in garden-based education: examining models and measures derived from self-determination theory. *J. Environ. Educ.* 43 16–36. 10.1080/00958964.2011.596856

[B81] Studente S. Seppala N. Sadowska N. (2016). Facilitating creative thinking in the classroom: investigating the effects of plants and the colour green on visual and verbal creativity. *Think. Skills Creat.* 19 1–8. 10.1016/j.tsc.2015.09.001

[B82] Swank J. M. Cheung C. Prikhidko A. Su Y.-W. (2017). Nature-based child-centered play therapy and behavioral concerns: a single-case design. *Int. J. Play Ther.* 26 47–57. 10.1037/pla0000031

[B83] Takayama N. Korpela K. Lee J. Morikawa T. Tsunetsugu Y. Park B. J. (2014). Emotional, restorative, and vitalizing effects of forest and urban environments at four sites in Japan. *Int. J. Environ. Res. Public Health* 11 7207–7230. 10.3390/ijerph110707207 25029496 PMC4113871

[B84] Taylor A. F. Wiley A. Kuo F. E. Sullivan W. C. (1998). Growing up in the inner city: green spaces as places to grow. *Environ. Behav.* 30 3–27. 10.1177/0013916598301001

[B85] Taylor G. Jungert T. Mageau G. A. Schattke K. Dedic H. Rosenfield S. (2014). A self-determination theory approach to predicting school achievement over time: the unique role of intrinsic motivation. *Contemp. Educ. Psychol.* 39 342–358. 10.1016/j.cedpsych.2014.08.002

[B86] Truong S. Gray T. Ward K. (2016). “Sowing and growing” life skills through garden-based learning to re-engage disengaged youth. *Learn. Landsc.* 10 361–385.

[B87] Ulset V. Vitaro F. Brendgren M. Bekkus M. Borge A. I. H. (2017). Time spent outdoors during preschool: links with children’s cognitive and behavioral development. *J. Environ. Psychol.* 52 69–80. 10.1016/j.jenvp.2017.05.007

[B88] van den Berg A. E. van den Berg C. G. (2011). A comparison of children with ADHD in a natural and built setting. *Child Care Health Dev.* 37 430–439. 10.1111/j.1365-2214.2010.01172.x 21143265

[B89] Warber S. L. DeHurdy A. A. Bialko M. F. Marselle M. R. Irvine K. N. (2015). Addressing “nature-deficit disorder”: a mixed methods pilot study of young adults attending a wilderness camp. *Evid. Based Complement. Altern. Med.* 2015 1–13. 10.1155/2015/651827 26788110 PMC4695668

[B90] Weinstein N. Przybylski A. K. Ryan R. M. (2009). Can nature make us more caring? Effects of immersion in nature on intrinsic aspirations and generosity. *Pers. Soc. Psychol. Bull.* 35 1315–1329. 10.1177/0146167209341649 19657048

[B91] Wells N. M. Evans G. W. (2003). Nearby nature: a buffer of life stress among rural children. *Environ. Behav.* 25:311 10.1177/0013916503035003001

[B92] Wells N. M. Myers B. M. Todd L. E. Barale K. Gaolach B. Ferenz G. (2015). The effects of school gardens on children’s science knowledge: a randomized controlled trial of low-income elementary schools. *Int. J. Sci. Educ.* 37 2858–2878. 10.1080/09500693.2015.1112048

[B93] Wessler S. L. (2003). Rebuilding classroom relationships – It’s hard to learn when you’re scared. *Educ. Leadersh.* 61 40–43. 19768809

[B94] White R. (2012). A sociocultural investigation of the efficacy of outdoor education to improve learning engagement. *Emot. Behav. Diffic.* 17 13–23. 10.1080/13632752.2012.652422

[B95] Wiens V. Kyngäs H. Pölkki T. (2016). The meaning of seasonal changes, nature, and animals for adolescent girls’ wellbeing in northern Finland. A qualitative descriptive study. *Int. J. Qual. Stud. Health Well-being* 11:30160. 10.3402/qhw.v11.30160 26905401 PMC4764957

[B96] Williams D. R. Dixon P. S. (2013). Impact of garden-based learning on academic outcomes in schools: synthesis of research between 1990 and 2010. *Rev. Educ. Res.* 83 211–235. 10.3102/0034654313475824

