# Supplementary material for: Do Experiences With Nature Promote Learning? Converging Evidence of a Cause-and-Effect Relationship
Source: Front Psychol. 2019 Feb 19;10:305. doi: 10.3389/fpsyg.2019.00305 (PMC6401598; doi:10.3389/fpsyg.2019.00305)
Supplement: Supplementary file 1 [file Table_1.pdf]

Supplemental Materials

Methods: Scope, Scale, & Procedure

We utilized several recent research summaries and systematic literature reviews to take a snapshot of the state of the research on nature's impact on learning at the time of their publication and to suggest possible pathways explaining nature's impact on learning. We found relatively little that articulated a coherent mechanistic model. We then engaged in a targeted literature review meant to provide an update on the themes evidenced in those existing research summaries and literature reviews, to fill in gaps we observed in the prior reviews, and to uncover literature to inform our attempt to articulate the pathways that might explain nature's impact on learning. The literature review process consisted of three main phases conducted in sequence.

Phase 1. The first step was to utilize recent peer-reviewed research summaries relevant to NBI and identify major themes related to NBI at the time of their publication. Summary articles that provided the basis for the review included: Berezowitz, Yoder, & Schoeller (2015), Bell & Dyment (2008), Blair (2009), Chawla (2015), Gill (2014), & Williams & Dixon (2013). The purpose of this phase was to understand the previous state of the literature and the main themes in the literature at the time of those reviews' publication. Articles referenced in these previous reviews were included in our database of articles.

Phase 2. The second step was to collect peer-reviewed journal articles that were published since the cut-off dates for previous reviews. This meant conducting a literature search starting with the most recent year and working backwards towards the date of the latest review (2015) so as not to overlap with articles included in previous reviews. This research was limited to articles published in English. There were no restrictions based on where a study was conducted nor on methodology. The review included work addressing any aspect of learning and developmental outcomes associated with any aspect of nature. A full list of keywords used in the search process is located in Table S2. At this time, the purpose was to update and expand findings from the previous review papers, and to get a sense for the diversity of the literature as a whole based on location, methodological approach, outcome variables, and sample type.

Phase 3. The third and last step was to examine studies bearing on likely mechanisms by which nature might impact learning, as well as studies on the effectiveness of the kinds of instructional approaches typically used in nature-based instruction. Likely mechanisms were identified by examining the nature benefits literature and, for each benefit, determining whether it was understood to be a precursor for learning (e.g., one of the benefits of nature experiences is improved concentration; concentration is, in turn, understood to be an important factor in learning). Descriptions of the instructional approaches used in nature-based instruction (e.g., student-centered, active learning, problem-oriented, collaborative, etc) served as the basis of a search of the educational psychology literature, to see if these approaches had been tested for their effectiveness compared to traditional instruction.

**Table S1. Operational Definitions of Key Terminology**
Nature: nature is any environment, built or natural, with natural elements, such as rocks, soil, sand, water, vegetation, animals, insects, and other natural materials and elements. Settings range from indoor classrooms incorporating a fish tank to city parks to gardens to native landscapes. Nature-based learning

occurs in a variety of contexts: in cities, towns, neighborhoods, wilderness areas, farms, rural settings, museums, classrooms, schools and any other places that afford such opportunities. In the review of nature-learning studies and nature-mechanism studies, we required that nature must be a central focus of the study; again, nature in a built space was included; mapping studies using GIS or satellite data were included; and studies involving images or views of nature were also included.
Learning: The acquisition and development of knowledge, attitudes, behaviors, values, and skills through experience, study, being taught and/or self-directed exploration. We recognize that learning involves component processes (e.g., attention and retention) which are related to other physical, emotional and cognitive processes (e.g., sense of agency, motivation, regulation, and engagement in learning). Learning was thus defined holistically to include more than simply the acquisition of knowledge. We also examined two broad categories of research bearing on the relationship between nature and learning, but which omitted either nature or learning: research on possible nature → learning mechanisms and research on "active ingredients" of the impacts of nature on learning. For example, the research on the impacts of nature on attention was reviewed as well as the research on the impacts of attention on learning because improved attention is a potential mechanism by which nature might impact learning. Similarly, we reviewed studies of two major components of nature-based instruction - the natural setting and the characteristics of the pedagogical approach that nature-based settings tend to afford — active, hands-on, collaborative, student-centered, etc. We reviewed evidence on the impacts of these pedagogical approaches on learning outside the context of natural settings. Further, we reviewed the impacts of nature in educational settings outside the context of nature-based instruction (i.e., instruction that does not explicitly incorporate nature as an element or focus of the lesson).
Academic Outcomes: Evidence of achievement, including, and not limited to, mastery of knowledge as demonstrated by academic performance on tests or by earning grades; educational attainment such as high school graduation; demonstration of skills such as math or language skills; measurable changes in attitudes toward learning; and performance of behaviors that contribute to learning such as school attendance or self-regulation. Educational outcomes may also include changes in attitudes and behaviors related to conservation ethics, care for nature, and environmental stewardship. Academic outcomes were defined broadly to be inclusive of simple achievement metrics to provide a more holistic perspective. This allowed not only for a more comprehensive look at the effects of NBI generally, but was also inclusive of educational outcomes across ages and experience types.

**Table S2. Master Literature Search Keywords**
General: place-based learning, nature-based learning, calm(er)*, quiet(er)*, safe(r)*, warm(er)*, cooperative*, cooperation*, intergroup relations*, loose parts*, learning context*
Nature: nature, natural, environment, garden, park, trees, green space, forest, farm, plants, wildlife, wilderness, outdoor(s), yard, classrooms, schoolyard, forest school, school gardens, outdoor learning environment, outdoor education, educational setting
Learning and components of learning: learning, education, study, experience, exploration, attention,

retention, stress, impulse control*, impulsivity*, inhibitory control*, creativity*, creative*, meta-cognition*, meta-cognitive*, metacognition*,
Educational Outcomes: attainment, grades, test scores, exams, graduation, college, achievement, intelligence, engagement, academic, GPA, ACT, SAT
** Denotes keywords that we're added in Phase 3 of the literature review process.*<br>*Note: keyword searches used combinations of the keywords listed above. Some keywords proved too broad to be meaningfully searched in the current context (e.g. nature), in which case narrower terms were substituted (e.g., "natural environment").*

As a service to the research and practice communities, we are making public a multi-layered spreadsheet providing information about articles retrieved in the process of this review: https://goo.gl/FZ1CA9.

Clarifications & Comments

Figure 1 & Table 1 in the manuscript reflect insights emerging from the literature review rather than conceptual frameworks that guided the review. Figure 1 shows mechanisms for which there is correlational evidence or better for both the link between nature and a given mechanism and between that mechanism and learning, personal development, or stewardship. Table 1 compiles advances made in the area of NBI generally from early work through the time of this writing, although the vast majority of progress has been quite recent. These advances do not come from any individual study in particular but rather reflect insights from across the body of literature.

Three topics we examined but ultimately chose not to include were: asthma, because although nature is related to asthma, the link between asthma and learning was unclear; obesity, because although nature is related to obesity, the link between obesity and learning is, again, unclear; and metacognition, because there has been a general lack of studies in this area despite some hints that nature may support the development of metacognitive skills (see Sproule et al., 2013; & Blair, 2009).
